# Nucleoside-modified mRNA immunization elicits influenza virus hemagglutinin stalk-specific antibodies

**DOI:** 10.1038/s41467-018-05482-0

**Published:** 2018-08-22

**Authors:** Norbert Pardi, Kaela Parkhouse, Ericka Kirkpatrick, Meagan McMahon, Seth J. Zost, Barbara L. Mui, Ying K. Tam, Katalin Karikó, Christopher J. Barbosa, Thomas D. Madden, Michael J. Hope, Florian Krammer, Scott E. Hensley, Drew Weissman

**Affiliations:** 1 Department of Medicine, University of Pennsylvania, Philadelphia, PA 19104 USA; 2 Department of Microbiology, Perelman School of Medicine, University of Pennsylvania, Philadelphia, PA 19104 USA; 3 Department of Microbiology, Icahn School of Medicine at Mount Sinai, New York, NY 10029 USA; 4 Graduate School of Biomedical Sciences, Icahn School of Medicine at Mount Sinai, New York, NY 10029 USA; 5 Acuitas Therapeutics, Vancouver, BC V6T 1Z3 Canada; 6 BioNTech RNA Pharmaceuticals, An der Goldgrube 12, 55131 Mainz, Germany

## Abstract

Currently available influenza virus vaccines have inadequate effectiveness and are reformulated annually due to viral antigenic drift. Thus, development of a vaccine that confers long-term protective immunity against antigenically distant influenza virus strains is urgently needed. The highly conserved influenza virus hemagglutinin (HA) stalk represents one of the potential targets of broadly protective/universal influenza virus vaccines. Here, we evaluate a potent broadly protective influenza virus vaccine candidate that uses nucleoside-modified and purified mRNA encoding full-length influenza virus HA formulated in lipid nanoparticles (LNPs). We demonstrate that immunization with HA mRNA-LNPs induces antibody responses against the HA stalk domain of influenza virus in mice, rabbits, and ferrets. The HA stalk-specific antibody response is associated with protection from homologous, heterologous, and heterosubtypic influenza virus infection in mice.

## Abstract (web summary)

The highly conserved influenza virus hemagglutinin (HA) stalk represents a potential target for a broadly protective vaccine. Here, the authors show that immunization with nucleoside-modified mRNA encoding full-length HA formulated in lipid nanoparticles elicits HA stalk-specific antibodies and protects from heterosubtypic virus infection.

## Introduction

Seasonal influenza virus epidemics pose a significant global health threat. Inactivated and live attenuated vaccines have limited effectiveness and need to be reformulated every year. These vaccines induce antibody responses primarily against the immunodominant globular head domain of influenza virus hemagglutinin (HA), but the virus can easily escape from protective immune responses due to the plasticity of the HA head (reviewed in ref. ^1^). It may be possible to induce broader protection with vaccines that target more conserved viral regions, such as the stalk domain of HA that is less tolerant of escape mutations (reviewed in ref. ^2^). In recent years, several HA immunogens have been developed that elicit HA stalk-specific immune responses. For example, headless HAs and chimeric HAs (cHAs) induce potent stalk-reactive antibodies^3–7^. Heterologous prime-boost immunizations were also shown to elicit HA stalk-specific antibodies in preclinical studies^6–11^. Notably, most HA stalk-based vaccines require multiple immunizations.

In vitro-transcribed messenger RNA (mRNA)-based vaccines have shown promise against cancer and infectious diseases (reviewed in ref. ^12^). For example, a lipid nanoparticle (LNP) encapsulated^13^ 1-methylpseudouridine-modified mRNA vaccine protected mice and non-human primates against Zika virus infection after a single low dose immunization^14^. Although, several recent studies indicate that mRNA-based vaccines can provide protection against influenza virus infection, none of these reports determined if mRNA-based influenza virus vaccines elicited broadly reactive antibodies capable of neutralizing antigenically distinct influenza virus strains after a single immunization^15–19^.

Here, we demonstrate that vaccination with influenza virus HA-encoding, nucleoside-modified^20^, and fast protein liquid chromatography (FPLC)-purified^21^ mRNA-LNPs induces potent antibody responses that target the conserved HA stalk domain in mice, rabbits, and ferrets. These broadly reactive antibody responses were associated with protection from homologous, heterologous, and heterosubtypic influenza viruses in mice. We propose that nucleoside-modified, FPLC-purified mRNA-LNP vaccines represent a promising broadly protective influenza virus vaccine candidate.

## Results

### HA head and stalk-specific antibody responses in mice

**Fig. 1:**
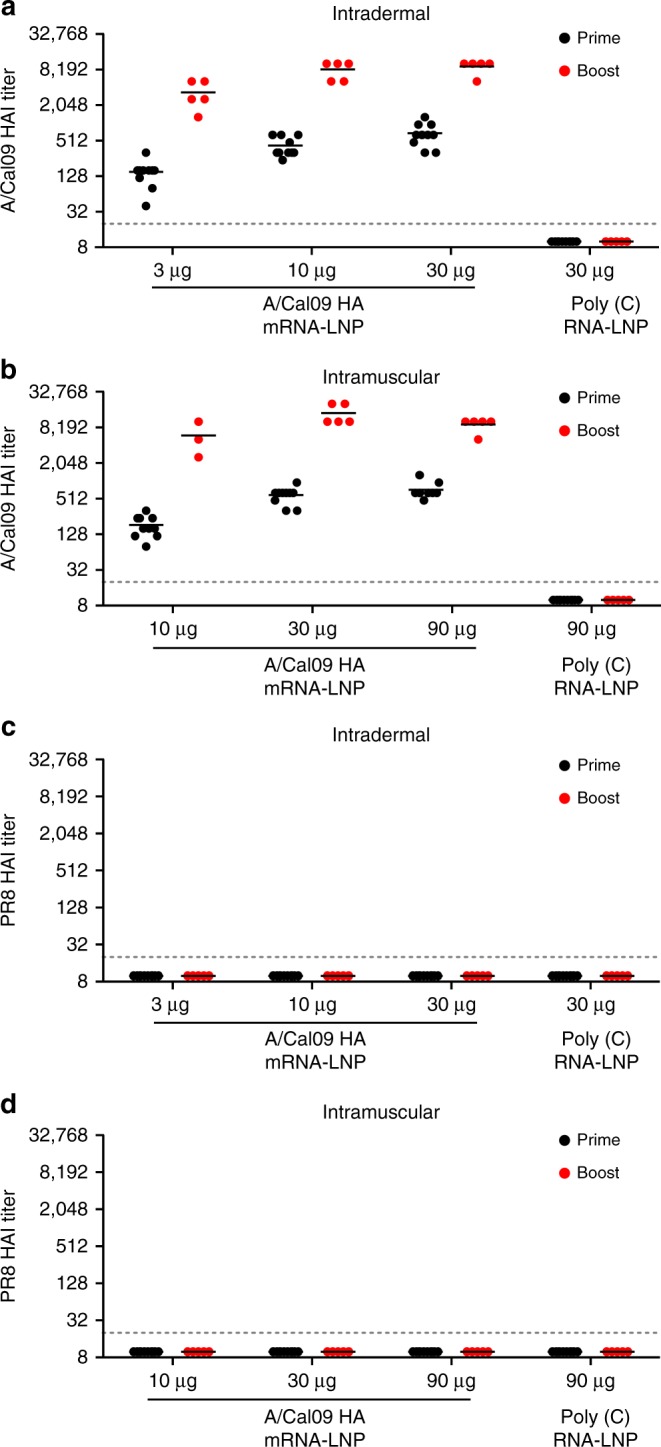
Nucleoside-modified HA mRNA-LNP immunization elicits potent neutralizing antibody responses in mice. Mice received two i.d. (3, 10, or 30 µg) or i.m. (10, 30, or 90 µg) immunizations of A/California/07/2009 HA mRNA-LNPs or 30 (i.d.) or 90 (i.m.) µg of poly(C) RNA-LNPs at week 0 (prime) and 4 (boost). HA inhibition (HAI) titers against A/California/07/2009 (**a**, **b**) and A/Puerto Rico/8/1934 (**c**, **d**) viruses were determined at week 4 and 8. *n* = 3–10 mice and each symbol represents one animal. Two independent experiments were performed. Horizontal lines show the mean; dotted line indicates the limit of detection. Statistical analysis: **a**, **b** two-way ANOVA with Bonferroni correction on log-transformed data, *p* < 0.05; all comparisons across doses and time points were statistically significant except for (**a**) 10 µg HA prime vs. 30 µg HA prime and 10 µg HA boost vs. 30 µg HA boost and for (**b**) 10 µg HA boost vs. 90 µg HA boost, 30 µg HA prime vs. 90 µg HA prime and 30 µg HA boost vs. 90 µg HA boost

To evaluate the immunogenicity of the nucleoside-modified HA mRNA-LNP vaccine, mice were immunized twice with 3, 10, or 30 µg of A/California/07/2009 (H1N1) (A/Cal09) HA-encoding mRNA-LNPs intradermally (i.d.) or 10, 30, or 90 µg of A/California/07/2009 HA mRNA-LNPs intramuscularly (i.m.) and antibody responses were assessed. The two immunizations were delivered 4 weeks apart. Control animals were vaccinated with 30 µg (i.d.) or 90 µg (i.m.) of poly(C) RNA-LNPs. A single immunization induced potent antibody responses targeting the HA globular head domain as determined by hemagglutination inhibition (HAI) assays using the homologous A/California/07/2009 virus (Fig. 1a, b). Higher vaccine doses elicited higher HAI titers with subtle differences between the i.d. and i.m.-immunized animals. A second immunization substantially increased HAI titers that reached 1:1280–1:20,480 at week 8 (Fig. 1a, b). As expected, antibodies elicited by the A/California/07/2009 HA mRNA-LNPs had no HAI activity against the A/Puerto Rico/8/1934 H1N1 virus (Fig. 1c, d), which possesses a genetically divergent HA globular head domain (Supplementary Fig. 1).

**Fig. 2:**
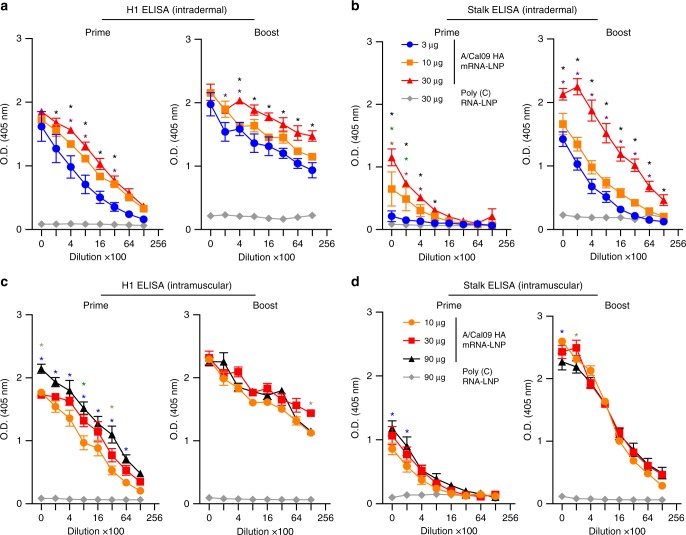
Nucleoside-modified HA mRNA-LNP immunization elicits HA stalk-specific antibody responses in mice. Mice received two i.d. (3, 10, or 30 µg) (**a**, **b**) or i.m. (10, 30, or 90 µg) (**c**, **d**) immunizations of A/California/07/2009 HA mRNA-LNPs or 30 (i.d.) or 90 (i.m.) µg of poly(C) RNA-LNPs at weeks 0 and 4. IgG binding to full-length H1 HA (**a**, **c**) and cH6/1 HA (**b**, **d**) proteins was determined at week 4 and week 8. *n* = 3–10 mice. Two independent experiments were performed. Error bars are SEM. Statistical analysis: **a**, **b** two-way ANOVA with Bonferroni correction, *p < 0.05 comparing A/California/07/2009 HA mRNA-LNP dose groups at each serum dilution, black star: significant difference in titers between the 3 and 30 µg groups, purple star: significant difference in titers between the 3 and 10 µg groups, green star: significant difference in titers between the 10 and 30 µg groups; **c**, **d** two-way ANOVA with Bonferroni correction, *p < 0.05 comparing A/California/07/2009 HA mRNA-LNP dose groups at each serum dilution, blue star: significant difference in titers between the 10 and 90 µg groups, green star: significant difference in titers between the 10 and 30 µg groups, gray star: significant difference in titers between the 30 and 90 µg groups

We next performed enzyme-linked immunosorbent assays (ELISAs) using cHA antigens to quantify HA stalk-reactive antibodies elicited by nucleoside-modified HA mRNA-LNP vaccination. For these experiments, we measured antibody binding to full-length A/California/07/2009 HA and an H6/1 cHA (cH6/1 HA) that possesses an H1 stalk domain and an H6 “exotic” globular head domain. Previous studies have shown that H1 stalk-reactive antibodies bind to both the full-length H1 construct and the cH6/1 construct, whereas H1 globular head-reactive antibodies bind only to the full-length H1 construct and not the cH6/1 construct^22^. Antibodies from all A/California/07/2009 HA-vaccinated mice showed very strong binding to full-length A/California/07/2009 HA after a single immunization (Fig. 2a, c). Importantly, HA stalk-reactive antibodies capable of binding to cH6/1 HA were also elicited by a single immunization. A booster immunization significantly increased HA stalk-reactive antibodies capable of binding to cH6/1 HA (Fig. 2b, d).

**Fig. 3:**
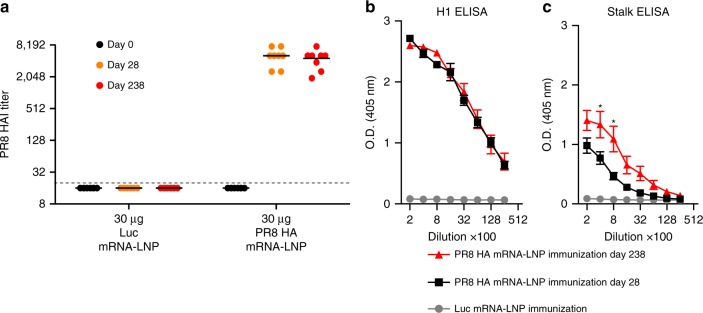
Nucleoside-modified HA mRNA-LNP immunization elicits durable HA stalk-specific antibody responses in mice. Mice received a single i.d. dose of 30 µg of A/Puerto Rico/8/1934 HA mRNA-LNP vaccine and **a** HAI titers against A/Puerto Rico/8/1934 and IgG binding to full-length H1 HA (**b**) and cH5/1 HA (**c**) proteins in mouse serum obtained 28 and 238 days post single immunization were determined. *n* = 8 mice. **a** Each symbol represents one animal, horizontal lines show the mean, dotted line indicates the limit of detection. **b**, **c** Error bars are SEM. Statistical analysis: **a** two-way ANOVA with Bonferroni correction on log-transformed data, *p* < 0.05; all comparisons between the Luc and the day 28 and day 238 Puerto Rico/8/1934 HA groups were statistically significant. **b**, **c** Two-way ANOVA with Bonferroni correction comparing Puerto Rico/8/1934 HA day 28 and day 238 time points for different dilutions. *p < 0.05

To determine if HA stalk-specific antibodies could be elicited with a different influenza HA immunogen, mice were i.d. immunized with a single dose of 30 µg of A/Puerto Rico/8/1934 (PR8) influenza HA mRNA-LNPs or poly(C) RNA-LNPs and HAI activity and HA stalk-specific antibody responses were followed over time. A single immunization elicited high HAI titers against the homologous A/Puerto Rico/8/1934 virus (Fig. 3a). Consistent with our results using mRNA-LNPs expressing A/California/07/2009 HA, A/Puerto Rico/8/1934 HA mRNA-LNPs elicited high levels of antibodies to full-length A/Puerto Rico/8/1934 HA and a significant portion of these antibodies recognized the HA stalk domain of a cH5/1 HA construct (Fig. 3b, c). We also tested the durability of antibody responses in this experiment, and found that anti-HA antibody levels remained unchanged over 30 weeks post-vaccination, and, in fact, the stalk responses were stronger compared to 4 weeks post-immunization (Fig. 3).

Durable, protective responses toward the HA require CD4^+^ T cell functions^23^, thus we examined CD4^+^ T cell responses after nucleoside-modified mRNA-LNP vaccination. Mice were immunized with a single 30 µg dose of A/California/07/2009 HA-encoding mRNA-LNPs i.d. and CD4^+^ T cell responses were determined 12 days later. As a comparator, a group of mice were immunized with 3 µg of monovalent A/California/07/2009 virus vaccine. Unlike inactivated virus immunization, HA mRNA-LNP vaccination induced HA-specific CD4^+^ T cell responses as measured by intracellular cytokine production (Supplementary Fig. 2).

### HA stalk-reactive antibody responses in rabbits and ferrets

To further evaluate the potency of the nucleoside-modified mRNA-LNP influenza virus vaccine, immunogenicity was also evaluated in rabbits and ferrets. Rabbits were i.d. immunized twice with 50 µg of A/California/07/2009 HA mRNA-LNPs and antibody responses were evaluated. A single immunization elicited HAI titers ranging from 1:120 to 1:320, as well as, HA stalk-specific antibody responses (Supplementary Fig. 3). A second immunization significantly boosted antibody responses and resulted in increased HAI titers and stalk-specific antibodies (Supplementary Fig. 3).

**Fig. 4:**
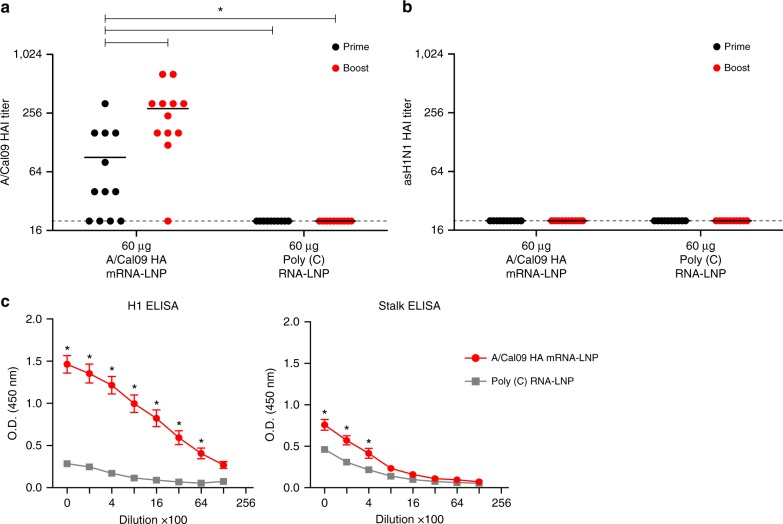
Nucleoside-modified HA mRNA-LNP immunization induces HA stalk-reactive antibodies in ferrets. Ferrets were immunized two times i.m. with 60 µg of A/California/07/2009 HA mRNA-LNPs or 60 µg of poly(C) RNA-LNPs at week 0 (prime) and 4 (boost). HAI titers against the A/California/07/2009 (**a**) and A/swine/Jiangsu/40/2011 (**b**) viruses were determined at week 4 (prime) and week 13 (boost). **c** IgG binding to full-length H1 HA (total) and cH6/1 HA (stalk) proteins from serum samples obtained 9 weeks after the second immunization was determined. *n* = 12 ferrets. **a**, **b** Each symbol represents one animal, horizontal lines show the mean, dotted line indicates the limit of detection. **c** error bars are SEM. Statistical analysis: **a**, **b** one-way ANOVA with Bonferroni correction on log-transformed data, *p < 0.05. **c** Two-way ANOVA with Bonferroni correction comparing A/California/07/2009 HA and poly(C) immunizations for different dilutions. *p < 0.05

Next, we evaluated the nucleoside-modified mRNA-LNP influenza virus vaccine in a ferret model. Animals were immunized i.m. twice with 60 µg of A/California/07/2009 HA mRNA-LNPs. Antibodies against the HA globular head domain were elicited in 8 out of 12 ferrets (≥1:40 HAI in all responders) 4 weeks after the first immunization as measured by HAI assays (Fig. 4a). Substantially increased HAI titers (≥1:160 in 10 out of 12 animals) were obtained after the boost. No HAI activity against the heterologous asH1N1 (A/swine/Jiangsu/40/2011, Supplementary Fig. 4) virus was detected (Fig. 4b). Importantly, animals generated HA stalk-reactive antibodies that bound to the cH6/1 HA protein (Fig. 4c). Moreover, as measured by in vitro microneutralization (MN) experiments, sera obtained from ferrets 4 weeks after the first and 9 weeks after the second immunization neutralized the closely related pH1N1 (A/Netherlands/602/2009) and the more antigenically distant asH1N1 virus (Supplementary Fig. 5).

Collectively, these studies indicate that nucleoside-modified HA mRNA-LNP vaccines elicit high levels of antibodies against both the HA head and stalk domains of influenza virus in mice, rabbits, and ferrets.

### Protection from homologous and heterologous viruses in mice

**Fig. 5:**
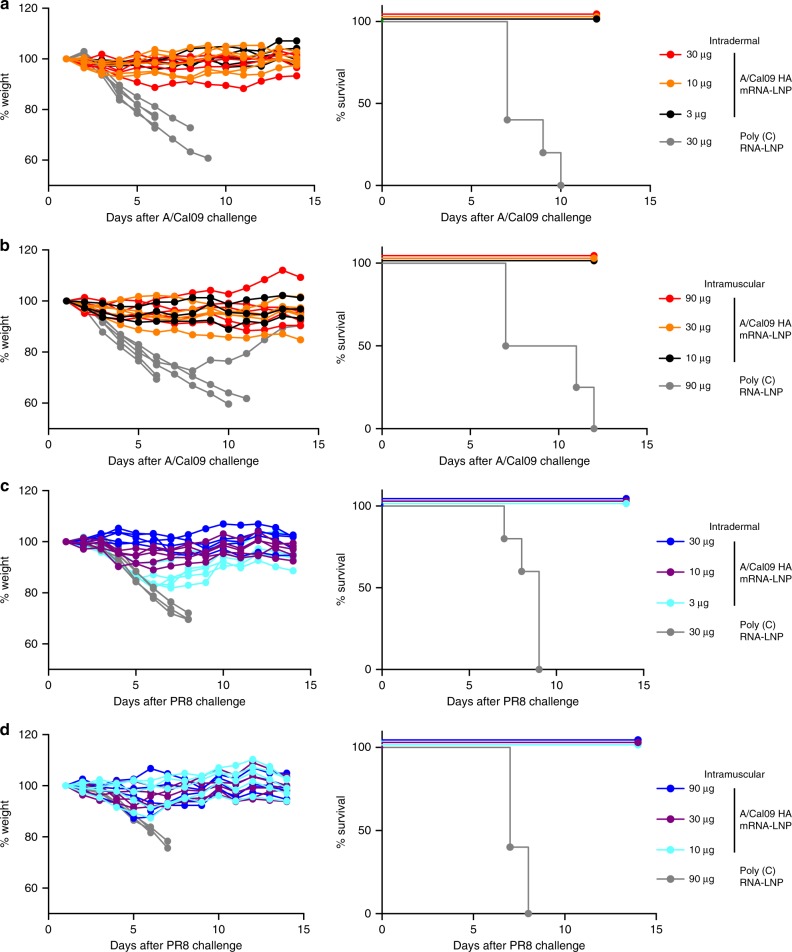
Two immunizations with nucleoside-modified A/California/07/2009 HA mRNA-LNP vaccine elicits protection from A/California/07/2009 and Puerto Rico/8/1934 viruses. Mice received two i.d. (3, 10, or 30 µg) or i.m. (10, 30, or 90 µg) immunizations of A/California/07/2009 HA mRNA-LNPs or 30 (i.d.) or 90 (i.m.) µg of poly(C) RNA-LNPs at week 0 (prime) and 4 (boost). Animals were challenged with lethal doses of homologous A/California/07/2009 (**a**, **b**) or heterologous A/Puerto Rico/8/1934 viruses (**c**, **d**) 5 weeks after the second immunization and weight loss and survival were followed. Two independent experiments were performed. *n* = 5 mice and each weight loss line represents one animal

To investigate the protective efficacy of mRNA immunization, mice that were immunized with two i.d. or i.m. doses of A/California/07/2009 HA mRNA-LNPs were challenged with the homologous A/California/07/2009 H1N1 virus or the heterologous A/Puerto Rico/8/1934 H1N1 virus 5 weeks after the last immunization. Both challenge viruses possessed A/Puerto Rico/8/1934 internal genes and the A/California/7/2009 HA possessed a D225G mutation to facilitate viral replication in mice. All HA mRNA-LNP-vaccinated animals were protected from both the homologous (Fig. 5a, b) and heterologous H1N1 virus infection (Fig. 5c, d), although some initial weight loss in the low dose groups was observed after challenge with the A/Puerto Rico/8/1934 virus (Fig. 5c). Mice injected with control poly(C) RNA-LNP lost weight and died or needed to be euthanized after viral challenge (Fig. 5).

**Fig. 6:**
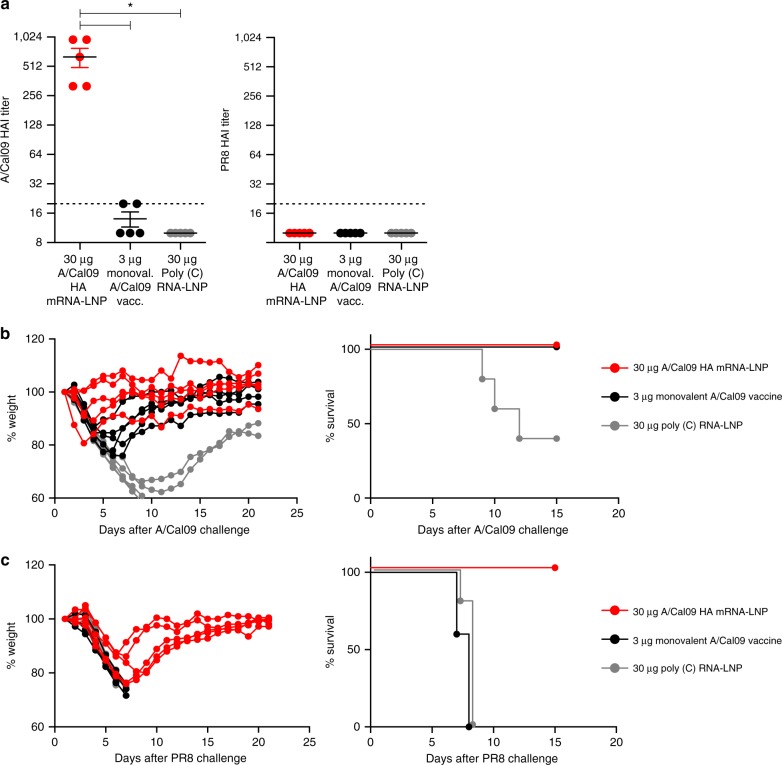
Nucleoside-modified A/California/07/2009 HA mRNA-LNP vaccine elicits protection from A/California/07/2009 and A/Puerto Rico/8/1934 viruses after a single immunization. Mice received a single i.m. dose of 30 µg A/California/07/2009 HA mRNA-LNPs. Control animals were vaccinated i.m. with a single dose of 3 µg of monovalent A/California/07/2009 virus vaccine or 30 µg of poly(C) RNA-LNPs. HAI titers against the A/California/07/2009 and A/Puerto Rico/8/1934 virus (**a**) were determined 28 days post single immunization. Animals were challenged with lethal doses of homologous A/California/07/2009 (**b**) or heterologous A/Puerto Rico/8/1934 (**c**) viruses 28 days after immunization and weight loss and survival was followed. *n* = *5* mice. **a** Horizontal lines show the mean; dotted line indicates the limit of detection. **b**, **c** Each weight loss line represents one animal. Statistical analysis: **a** one-way ANOVA with Bonferroni correction on log-transformed data, *p < 0.05; **b** two-way ANOVA with Bonferroni correction on weight loss graphs comparing A/California/07/2009 mRNA-immunized animals to inactivated virus-immunized animals. *p* < 0.05 on days 4–6

Based on the high HAI titers and measurable HA stalk-reactive antibody responses elicited by the mRNA-LNP constructs, we hypothesized that a single HA mRNA-LNP immunization could elicit protective immunity. Mice were i.m. immunized with a single dose of 30 µg of A/California/07/2009 HA mRNA-LNPs or 30 µg of poly(C) RNA-LNPs. As a comparator, a group of mice were immunized with 3 µg of monovalent A/California/07/2009 virus vaccine. Four weeks after vaccination, mRNA-LNP vaccines induced HAI titers ranging between 320–960 against the autologous strain, whereas the monovalent virus vaccine elicited very low HAI titers of ~1:15 (Fig. 6a). No HAI activity against the PR8 virus was detected (Fig. 6a). High antibody titers elicited by the mRNA-LNP vaccine were associated with protection following challenge with the homologous A/California/07/2009 viral strain and the heterologous A/Puerto Rico/8/1934 viral strain (Fig. 6b, c). All mRNA-LNP-immunized and monovalent virus vaccine-injected animals survived A/California/07/2009 virus infection; however, animals in both groups lost a substantial amount of weight following infection (Fig. 6b). mRNA-LNP-immunized animals displayed weight loss following A/Puerto Rico/8/1934 virus challenge, but they rapidly recovered and survived virus infection (Fig. 6c). In contrast, mice that were immunized with the monovalent A/California/07/2009 virus vaccine or the poly(C) RNA-LNP vaccine rapidly developed symptoms and 100% of the animals died following A/Puerto Rico/8/1934 virus challenge (Fig. 6c). These data demonstrate that a single mRNA-LNP immunization induced protection from an antigenically distant H1 virus (Supplementary Fig. 1) in the absence of HAI activity to this viral HA.

### Protection from a heterosubtypic virus in mice

**Fig. 7:**
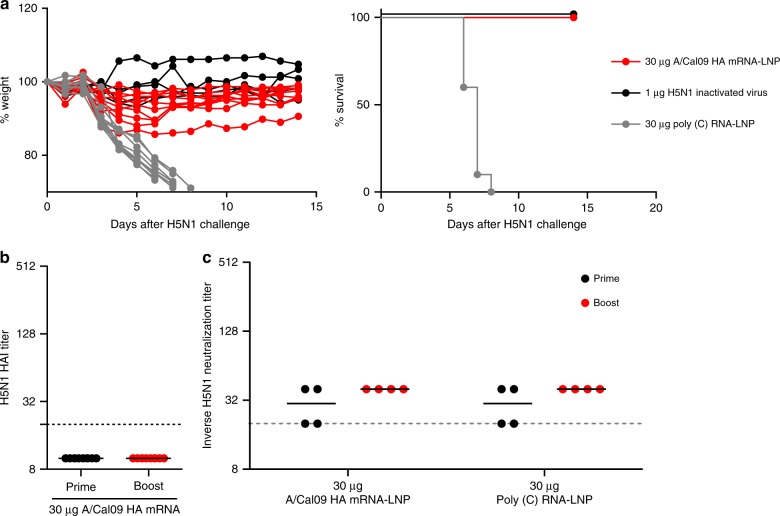
Nucleoside-modified A/California/07/2009 HA mRNA-LNP vaccine elicits protection from the A/Vietnam/1203/04 (H5N1) virus after two immunizations. Mice received two i.d. doses of 30 µg of A/California/07/2009 HA mRNA-LNPs at weeks 0 and 4. Control animals were vaccinated with two i.d. doses of 30 µg of poly(C) RNA-LNPs or two i.m. doses of 1 µg of inactivated H5N1 virus. (**a**) Animals were challenged with a lethal dose of A/Vietnam/1203/04 virus 28 days after the second immunization and weight loss and survival were followed. *n* = 10 mice. HAI titers (**b**) and in vitro microneutralization activity (**c**) against the A/Vietnam/1203/04 virus were determined 28 days after the first and 28 days after the second immunization. Pooled serum samples were used for MN assays. **a** Each weight loss line represents one animal. **b**, **c** Horizontal lines show the mean; dotted line indicates the limit of detection. Statistical analysis: **a** two-way ANOVA with Bonferroni correction on weight loss graphs comparing A/California/07/2009 mRNA-immunized animals to inactivated virus-immunized animals, *p* < 0.05 on days 5–7. **b** Unpaired *t*-test comparing post prime and post boost samples, *p < 0.05. **c** Two-way ANOVA with Bonferroni correction, *p < 0.05

We next performed a series of studies with an H5N1 influenza virus to determine whether nucleoside-modified HA mRNA-LNP immunization elicits protective immune responses against more antigenically distant influenza virus subtypes. Mice were immunized twice with 30 µg of A/California/07/2009 HA mRNA-LNPs i.d. or 1 µg (total protein) of inactivated H5N1 influenza virus i.m.^24^. Mice were then challenged with a lethal dose of H5N1 virus (6:2 reassortant between A/Puerto Rico/8/1934 and A/Vietnam/1203/04, which donated HA and neuraminidase) 4 weeks after the second immunization and weight and morbidity were monitored for 14 days. Strikingly, all HA mRNA-LNP-immunized animals survived viral challenge, while those injected with poly(C) RNA-LNP needed to be euthanized (Fig. 7a). As expected, no measurable HAI activity against the H5N1 virus was observed in mice immunized with the A/California/07/2009 HA mRNA-LNP vaccine (Fig. 7b). Interestingly, sera from these animals also did not display neutralization activity against the H5N1 virus in in vitro MN experiments (Fig. 7c).

## Discussion

Currently approved influenza virus vaccines offer protection against well-matched circulating strains. These regimens mostly elicit antibody responses against the continuously changing immunodominant globular HA head domain. There is a need to develop “universal” influenza virus vaccines that induce potent immune responses against conserved viral epitopes and offer protection from heterologous and heterosubtypic strains. There are several viral protein regions that are conserved among different influenza virus strains, including epitopes in the HA stalk domain, neuraminidase, the ectodomain of the ion channel M2, matrix protein, and nucleoprotein (reviewed in refs. ^25,26^). Vaccines against these targets might be able to elicit broad and protective influenza virus-specific immune responses (reviewed in ref. ^1^). One of the most extensively studied targets is the immunosubdominant HA stalk (reviewed in ref. ^27^). Seasonal influenza vaccines elicit poor antibody responses against HA stalk^28,29^ in most individuals; thus, the development of new vaccine platforms and optimized immunogens that specifically elicit antibodies against this region is critically important.

Previous studies, using stable, headless HA proteins^3,4^ or cHA-encoding DNA and proteins (prime-boost)^6^ demonstrated that HA stalk-specific antibodies can be elicited in mice and ferrets. Yassine and colleagues developed headless H1 HA immunogens and demonstrated that three immunizations elicited protective antibodies against H5N1 influenza virus in mice and ferrets^3^. Impagliazzo and coworkers generated various monomeric, dimeric, and trimeric headless H1 mini HAs and showed that three immunizations with the best working trimeric construct resulted in protection against an H5N1 virus in mice^4^, and potent antibody responses in cynomolgus monkeys. Ermler and colleagues designed cHAs with HA heads from various influenza A viruses and HA stalks from influenza B viruses^6^. Sequential vaccination (one DNA prime and two protein boosts) with these constructs resulted in protection from influenza B viruses in mice. A recent study from the same laboratory used sequential immunization in ferrets and demonstrated protection from pandemic H1N1 virus challenge^7^.

Here, we evaluated the efficacy of a newly developed influenza virus vaccine using nucleoside-modified, FPLC-purified full-length HA-encoding mRNA encapsulated in LNPs^30^. A single immunization with 3 µg of A/California/07/2009 HA mRNA-LNPs resulted in ≥1:120 A/California/07/2009 HAI titers at 4 weeks post-immunization in mice. A second dose induced HAI values ranging between 1280–20,480 depending on the dose and the route of delivery (Fig. 1a, b). Of note, vaccination with A/California/07/2009 mRNA-LNPs did not generate HAI activity against the A/Puerto Rico/8/1934 H1N1 influenza virus (Fig. 1c, d); however, the vaccine protected animals from A/Puerto Rico/8/1934 influenza virus challenge (Fig. 5c, d). This protection was likely mediated by HA stalk-reactive antibody responses that were measurable after a single immunization, and which were boosted by a subsequent immunization (Fig. 2).

A critical finding of this report is that a single immunization with A/California/07/2009 HA mRNA-LNPs resulted in protection against the homologous A/California/07/2009 and the heterologous A/Puerto Rico/8/1934 virus challenge in mice (Fig. 6b, c). Furthermore, we found that two immunizations with the A/California/07/2009 HA mRNA-LNP vaccine induced protection against H5N1 influenza virus infection (Fig. 7a). Of note, sera from these animals did not neutralize the challenge virus in in vitro MN experiments (Fig. 7c), which suggests a contribution of other effector immune mechanisms to heterosubtypic vaccine protection. These findings are in accordance with the literature as several recent studies demonstrated that the potency of HA stalk-specific antibodies were often enhanced by Fc receptor-mediated mechanisms such as antibody-dependent cell-mediated cytotoxicity or complement-dependent cytotoxicity^31–34^. Future studies will determine the correlates of protection after mRNA-LNP influenza vaccine administration. Several mRNA-based influenza vaccines have been described in recent years (reviewed in ref. ^35^). For example, Bahl and colleagues demonstrated that a similar mRNA-LNP vaccine platform (using nucleoside-modified but not FPLC-purified H7 and H10 HA mRNAs) induced protection against the homologous influenza virus after a single dose immunization in mice and ferrets; however, no heterologous challenge data or evidence for the presence of stalk-specific antibodies was evaluated in these studies^18^. Another recent report using unmodified HA mRNA-LNPs demonstrated durable induction of HA-specific antibodies in non-human primates, but again, the generation of cross-protective HA stalk antibodies was not reported^36^.

Ferrets are one of the best animal models for influenza virus research; however, a potential limitation of the use of ferrets for antiserum generation is that they usually generate more focused antibody responses to the variable head regions of HA than humans after natural infection^37,38^. We demonstrated that a single i.m. immunization with the A/California/07/2009 mRNA-LNP vaccine elicited ≥1:40 HAI activity against the A/California/07/2009 virus in 8 out of 12 animals 4 weeks after vaccination (Fig. 4a). A subsequent immunization resulted in ≥1:160 HAI titers in 10 out of 12 A/California/07/2009 HA mRNA-LNP-immunized ferrets. The high variability in HAI activity may be due to the genetic heterogeneity of the out-bred animals, and thus it is possible that in some ferrets mRNA-LNP immunization skewed immune responses toward epitopes that neutralize viruses but do not induce HAI titers (Fig. 4a). The presence of stalk-reactive antibodies (Fig. 4c) and MN titers against the homologous and heterologous H1N1 strains in A/California/07/2009 HA mRNA-LNP-immunized animals (Supplementary Fig. 5) support this hypothesis. Additionally, rabbits were immunized with the A/California/07/2009 HA mRNA-LNP vaccine and found that a single immunization elicited HA stalk-reactive antibodies (Supplementary Fig. 3), demonstrating that our findings in mice were translated to two species of large animals.

Here, we demonstrate that the nucleoside-modified, FPLC-purified influenza virus HA-encoding mRNA-LNP vaccine elicited HA stalk-specific antibody responses in mice, rabbits, and ferrets with durable HA stalk titers in mice. Future studies will address if mRNA-LNP immunization preferentially induce HA stalk-specific responses or they are elicited as a consequence of the very potent immune response without refocusing the immunodominance. Additional studies using adoptive serum transfer and T cell depletions will further evaluate the contribution of stalk-specific antibodies to vaccine protection in the heterosubtypic influenza virus infection model. The mRNA-LNP vaccine platform has additional beneficial features over other vaccines, including a favorable safety profile and highly scalable and potentially inexpensive manufacture. In addition to potency, from an influenza virus vaccine perspective, the most critical advantages of the present platform are the rapid development and the ease of production. It is notable that these vaccines are not subject to cell culture and egg-adaptive mutations that commonly arise as conventional influenza virus vaccines are manufactured^39,40^. The use of the nucleoside-modified mRNA-LNP platform with optimized HA stalk-inducing immunogens^3,4,7^ may offer a superior platform with easy clinical use. Currently available seasonal influenza virus vaccines do not protect well against antigenically drifted viral strains and they likely provide very little protection against emerging pandemic strains. Production of conventional, FDA-approved vaccines against new pandemic viruses could take at least 6 months, leaving the population unprotected during this period^1^. On the contrary, once the genetic sequence of the target HA antigen is known, mRNA-LNP vaccines can potentially be produced within weeks (reviewed in ref. ^12^). mRNA-LNP vaccine production is sequence-independent and can be applied to virtually any pathogen. We believe that the data presented in this report combined with the additional beneficial features of nucleoside-modified and purified mRNA-LNPs makes the present platform a viable broadly protective influenza virus vaccine candidate.

## Methods

### Ethics statement

The investigators faithfully adhered to the “Guide for the Care and Use of Laboratory Animals” by the Committee on Care of Laboratory Animal Resources Commission on Life Sciences, National Research Council. The animal facilities at the University of Pennsylvania, The Wistar Institute, the Icahn School of Medicine at Mount Sinai and Noble Life Sciences vivarium are fully accredited by the American Association for Accreditation of Laboratory Animal Care. All studies were conducted under protocols approved by the University of Pennsylvania, Noble Life Sciences, the Icahn School of Medicine at Mount Sinai, and The Wistar Institute IACUCs. The Wistar IACUC does not use weight loss as a criteria for euthanasia in murine influenza virus experiments.

### mRNA production

mRNAs were produced as previously described^41^ using T7 RNA polymerase (Megascript, Ambion) on linearized plasmids (synthesized by GenScript) encoding codon-optimized^42^ Puerto Rico/8/1934 influenza virus HA (pTEV-PR8 HA-A101), A/California/7/2009 influenza virus hemmaglutinin (pTEV-A/Cal09 HA-A101), and firefly luciferase (pTEV-Luc-A101). mRNAs were transcribed to contain 101 nucleotide-long poly(A) tails. One-methylpseudouridine (m1Ψ)-5′-triphosphate (TriLink) instead of UTP was used to generate modified nucleoside-containing mRNA. RNAs were capped using the m7G capping kit with 2′-*O*-methyltransferase (ScriptCap, CellScript) to obtain cap1. mRNA was purified by FPLC (Akta Purifier, GE Healthcare), as described^43^. All mRNAs were analyzed by denaturing or native agarose gel electrophoresis and were stored frozen at −20 °C.

### LNP formulation of the mRNA

FPLC-purified m1Ψ-containing firefly luciferase and influenza virus HA-encoding mRNAs and poly(C) RNA (Sigma) were encapsulated in LNPs using a self-assembly process in which an aqueous solution of mRNA at pH = 4.0 is rapidly mixed with a solution of lipids dissolved in ethanol^44^. LNPs used in this study were similar in composition to those described previously^44,45^, which contain an ionizable cationic lipid (proprietary to Acuitas)/phosphatidylcholine/cholesterol/PEG-lipid (50:10:38.5:1.5 mol/mol) and were encapsulated at an RNA to total lipid ratio of ~0.05 (wt/wt). They had a diameter of ~80 nm as measured by dynamic light scattering using a Zetasizer Nano ZS (Malvern Instruments Ltd., Malvern, UK) instrument. mRNA-LNP formulations were stored at −80 °C at a concentration of mRNA of ~1 μg/μl.

### Immunization of mice, rabbits, and ferrets

Mice: Female BALB/c mice aged 8 weeks were purchased from Charles River Laboratories. mRNA-LNPs were diluted in phosphate-buffered saline (PBS) and injected into animals intradermally with a 3/10cc 29½G insulin syringe (BD Biosciences). Four sites of injection (30 µl each) over the lower back were used. For intramuscular injections, mRNA-LNPs were diluted in PBS and injected into animals using a 3/10cc 29½G insulin syringe. Monovalent A/California/7/2009 virus vaccine (3 µg in 50 µl) (BEI Resources, NR-20347) and inactivated A/Vietnam/1203/04 virus vaccine (1 µg in 50 µl) (the virus was grown in eggs and inactivated with 0.3% formalin) were intramuscularly injected into the quadriceps muscle of animals using a 3/10cc 29½G insulin syringe. Inactivated H5N1 whole-virus vaccine preparation was generated by concentration of A/Vietnam/1203/04 virus (vaccine strain, 6:2 re-assortant with PR8, polybasic cleavage site of HA was removed) via ultracentrifugation followed by inactivation with 0.03% formalin. Protein content was measured using the Bradford method and reflects total protein rather than HA concentration.

Rabbits: Female New Zealand White rabbits aged 6 weeks were purchased from Charles River Laboratories. mRNA-LNPs were diluted in PBS and injected into animals intradermally with a 3/10cc 29G syringe (BD Biosciences). Six sites of injection (45 µl each) over the lower back were used.

Ferrets: 7–10-week-old male and female ferrets were purchased from Triple F Farms Inc. mRNA-LNPs were diluted in PBS and injected intramuscularly into the upper thigh (100 µl).

### Blood collection

Mice: Blood was collected prior to each immunization by submandibular bleeding. Blood was centrifuged for 10 min at 2000 × *g* in an Eppendorf microcentrifuge and the serum was stored at −80 °C and used for ELISA, MN, and HAI assays.

Rabbits: Blood was obtained from the lateral saphenous vein under anesthesia. Blood was centrifuged for 10 min at 3000 × *g* and the serum was stored at −80 °C and used for ELISA and HAI assays.

Ferrets: Blood was obtained from the vena cava under anesthesia. Blood was centrifuged for 10 min at 3000 × *g* and the serum was stored at −80 °C and used for ELISA, MN, and HAI assays.

### Antibody reagents for flow cytometry

The following antibodies were used for flow cytometry: anti-CD4 PerCP/Cy5.5 (Clone GK1.5, BioLegend), anti-CD3 APC-Cy7 (Clone 145-2C11, BD Biosciences), anti-TNF-α PE-Cy7 (Clone MP6-XT22, BD Biosciences), anti-IFN-γ AF700 (Clone XMG1.2, BD Biosciences), anti-IL-2 APC (Clone JES6-5H4, BD Biosciences).

### Mouse splenocyte stimulation/staining and flow cytometry

Single cell suspensions from mouse spleens were made in complete medium. Splenocytes were washed once in PBS and resuspended in complete medium at 2 × 10^7^ cells/ml. 2 × 10^6^ cells (100 µl) per sample were stimulated for 6 h at 37 °C using two overlapping influenza virus HA (A/California/07/2009) peptide pools (peptides are 14-mers or 15-mers, with 11 amino acid overlaps, provided by BEI resources, NR-19244) at 2 µg/ml per peptide. Golgi Plug (brefeldin A, BD Biosciences) and Golgi Stop (monensin, BD Biosciences) were diluted 1:100 and 1:143 in complete medium, respectively, and 20 µl from both were added to each sample to inhibit the secretion of intracellular cytokines after 1 h of stimulation. A PMA (10 ng/ml)-ionomycin (250 ng/ml) (Sigma) stimulated sample and unstimulated samples for each animal were included.

After stimulation, cells were washed in PBS and stained using the LIVE/DEAD Fixable Aqua Dead Cell Stain Kit and then surface stained for CD4 (2 µg/ml). Antibodies were incubated with cells for 30 min at RT. Following surface staining, cells were washed in FACS buffer and fixed using the Cytofix/Cytoperm kit (BD Biosciences), according to the manufacturer’s instructions. Following fixation, the cells were washed in the permeabilization buffer and incubated with antibodies against CD3 (1.6 µg/ml), TNF-α (1.6 µg/ml), IFN-γ (4 µg/ml), and IL-2 (4 µg/ml) for 1 h at RT. Following staining, the cells were washed with the permeabilization buffer, fixed (PBS containing 1% paraformaldehyde) and stored at 4 °C until analysis. Splenocytes were analyzed on a modified LSR II flow cytometer (BD Biosciences).

### Recombinant influenza virus cHAs

Recombinant HAs including H1 HAs of A/California/04/09 and A/Puerto Rico/8/34 and chimeric cH6/1_Calss_ (an H6 head domain from A/mallard/Sweden/81/02 on top of an H1 stalk domain from A/California/04/09 with a stabilizing mutation in the stalk domain) and cH5/1_PR8_ (an H5 head domain from A/Vietnam/1203/04 on top of an H1 stalk domain from A/Puerto Rico/8/34) constructs were expressed in the baculovirus expression system, as previously described^46–48^. They were then purified via a hexahistidine tag and Ni-nitrilotriacetic acid (NTA) resin.

### Influenza virus challenge studies

Mice were anesthetized with isoflurane and intranasally challenged with 200,000 TCID_50_ of A/California/07/2009 influenza virus (6:2 virus with 6 A/Puerto Rico/8/1934 internal genes and A/California/07/2009 HA and NA) or 5000 TCID_50_ of A/Puerto Rico/8/1934 influenza virus in 50 µl PBS. The A/California/7/2009 HA possessed a D225G mutation to facilitate viral replication in mice.

H5N1 challenges were performed with a 6:2 A/Vietnam/1203/04 reassortant virus (6 A/Puerto Rico/8/1934 internal genes and A/Vietnam/1203/04 HA and NA with the polybasic cleavage site removed from the HA). Animals were anesthetized using ketamine/xylazine (0.15 mg ketamine and 0.03 mg xylazine) delivered intraperitoneally in a volume of 100 µl. The mice were then inoculated with 50 µl of a virus dilution (in PBS) containing 5 murine 50% lethal doses (mLD_50_) of virus.

### Enzyme-linked immunosorbent assays

ELISA plates were coated overnight at 4 °C with 3 µg/ml of recombinant protein. ELISA plates were blocked with a 3% (w/vol) bovine serum albumin (BSA) solution in PBS for 2 h. Plates were then washed three times with PBS-Tween20 (0.1%) and serial dilutions of sera (diluted in 1% BSA in PBS) were added to the plates. After 2 h of incubation, plates were washed and alkaline phosphatase-conjugated or horseradish peroxidase (HRP)-conjugated secondary antibodies were added. After a 1 h incubation, plates were washed and a p-nitrophenyl phosphate or a 3,3′,5,5′-tetramethylbenzidine (TMB) substrate (Seracare, product number 50-00-03) was added. For HRP-based ELISAs, HCl was then added to stop the TMB reaction, and absorbance at 405 nm was measured using a plate reader. Results were obtained from technical duplicates.

### HAI assays

A/Puerto Rico/8/1934 and A/California/07/2009 HAI assays: Mouse sera were heat-treated for 30 min at 55 °C. Ferret sera were treated with receptor destroying enzyme (RDE) from *Vibrio cholerae* (Denka Seiken, Chuo-ku, Tokyo, Japan) for 2 h at 37 °C and then heat-treated for 30 min at 55 °C. Rabbit sera were RDE-treated for 2 h at 37 °C, heat-treated for 30 min at 55 °C, and then absorbed with turkey erythrocytes. Titrations were performed in 96-well round bottom plates (BD Biosciences). First, 5 µl of sera were added to 95 µl of PBS (1:20 dilution), then two-fold serial dilutions were performed up to 1:2560 in a volume of 50 µl. Next, four agglutinating doses of virus were added to a total volume of 100 µl. Finally, 12.5 µl of turkey erythrocytes (Lampire) (2% (vol/vol) solution) was added to each well, and mixed thoroughly. Agglutination was read after incubating for 1 h at RT. HAI titers were expressed as the inverse of the highest dilution that inhibited four agglutinating doses of influenza virus.

A/swine/Jiangsu/40/2011 and A/Vietnam/1203/04 HAI assays: Mouse and ferret sera were treated with three volumes (based on original sera volume) of RDE for 18 h at 37 °C. Three volumes (based on original serum volume) of 2.5% sodium citrate solution was then added to the RDE-treated serum samples and were then incubated at 56 °C for 1 h. Three volumes of PBS (based on original serum volume) were added to each sample for a final dilution of 1:10. Titrations were performed in 96-well round bottom plates (BD Biosciences). First, 50 µl of RDE-treated serum was added to the first well, then two-fold serial dilutions were performed up to 1:2048. Next, eight agglutinating doses of virus were added to a total volume of 50 µl. Virus and sera were then incubated at RT for 30 min with shaking. Following this incubation, 50 µl of chicken erythrocytes (Charles River Laboratories) (0.5% (vol/vol) solution) was added to each well, and mixed thoroughly. Agglutination was read after incubating for 1 h at 4 °C. HAI titers were expressed as the inverse of the highest dilution that inhibited four agglutinating doses of influenza virus.

All samples were run in at least technical duplicates. The challenge virus strains (detailed in “Influenza virus challenge studies”) were used for HAI assays.

### MN assays

Mouse and ferret sera were treated with three volumes (based on original sera volume) of RDE for 18 h at 37 °C. Three volumes (based on original serum volume) of 2.5% sodium citrate solution was then added to the RDE-treated serum samples and were then incubated at 56 °C for 1 h. Three volumes of PBS (based on original serum volume) were added to each sample for a final dilution of 1:10.

Madin–Darby Canine Kidney (MDCK) cells (ATCC number PTA-6500) maintained in complete Dulbecco’s Modified Eagle Medium with the addition of 10% fetal bovine serum, 1% Pen/Strep, 1% of a 1 M HEPES stock solution were plated at a concentration of 1.5 × 10^4^ cells per well in a 96-well plate and left to grow overnight at 37 °C, 5% CO_2_ until they reached 80–90% confluency.

RDE-treated sera was serially diluted two-fold in 1× minimal essential medium (MEM; 10% 10× MEM, 1% 200 mM L-glutamine, 1.6% of a 7.5% sodium bicarbonate stock solution (pH 7.5), 1% of a 1 M 4-(2-hydroxyethyl)-1-piperazineethanesulfonic acid (HEPES) stock solution, 1% of penicillin/streptomycin antibiotic cocktail (Pen/Strep, Gibco), 0.6% of a 35% BSA stock solution) containing 1 µg/ml L-1-tosylamide-2-phenylethyl chloromethyl ketone (TPCK)-treated trypsin. For ferret sera, half of the volume of the serial dilutions was incubated with 100×TCID_50_ of each virus (at a 1:1 volume ratio) for 1 h at RT with shaking. The virus-sera mixture was then applied to 80–90% confluent MDCK cells after they had been washed one time with sterile, 1×PBS. The cells were then incubated with the virus-sera mixture for 1 h at 33 °C. After the incubation, the virus-ferret sera mixture was removed, cells were washed with 1×PBS, and then covered with the remaining half of the serial sera dilutions (supplemented with a 1:1 addition of 1×MEM with 1 µg/ml TPCK-treated trypsin).

For mouse sera, the entire amount of sera was incubated with 50×TCID_50_ of H5N1 (at a 1:1 ratio) for 1 h at RT with shaking. The virus-sera mixture was then applied to 80–90% confluent MDCK cells after they had been washed one time with sterile 1×PBS. The cells were then incubated with the virus-sera mixture for 3 days at 33 °C. The difference in incubation time for the ferret sera vs. mouse sera on MDCK cells in these assays was due to the limited volumes of sera from mouse studies.

All samples were run in at least technical duplicates.

### Multiple sequence alignment and phylogenetic tree

Sequences of the full-length HA protein of A/California/04/2009 (pH1N1), A/Michigan/45/2015, A/New Caledonia/20/1999, A/South Carolina/1/1918, A/swine/Jiangsu/40/2011 (asH1N1), A/Brisbane/59/2007, A/swine/4/Mexico/2009, A/swine/Aichi/10/2015, A/swine/Guangxi/QZ5/2014, and A/swine/Ohio/A02216472/2017 were downloaded from the Global Initiative for Sharing of all Influenza Data (GISAID) and aligned using the online server, Clustal Omega (https://www.ebi.ac.uk/Tools/msa/clustalo/). The phylogenetic tree produced from this alignment was downloaded, rooted to A/South Carolina/1/1918, and visualized using FigTree.

### Mapping conservation of pH1N1 and asH1N1

The structure used for visualizing the conserved residues between pH1N1 and asH1N1 is PDB ID 3LZG^49^. An alignment of pH1N1 and asH1N1 whole-HA protein sequences (downloaded from GISAID) was used to determine 100% conserved residues in Chimeria v1.12. These residues were then mapped onto a monomer of 3LZG in red. The alignment of pH1N1 and asH1N1 was also uploaded to the LALIGN server (https://embnet.vital-it.ch/software/LALIGN_form.html) to determine percent identity and similarity between the two HA proteins.

### Statistical analyses

Statistical analyses were performed with Excel and Prism 5.0f software. Data were compared using one-way and two-way ANOVA corrected for multiple comparisons (Bonferroni method) and unpaired *t-*test. Survival analyses were perfomed using the log-rank (Mantel–Cox) test. The A/California/07/2009 crystal structure (PDB ID 3UBN) was visualized using PyMOL software.

### Data availability

All data are available within the article and its Supplementary Information file or from the authors upon request.

## Electronic supplementary material

Supplementary Information
