# Supplementary material for: Nucleoside-modified mRNA immunization elicits influenza virus hemagglutinin stalk-specific antibodies
Source: Nat Commun. 2018 Aug 22;9:3361. doi: 10.1038/s41467-018-05482-0 (PMC6105651; doi:10.1038/s41467-018-05482-0)
Supplement: Supplementary file 1 — Supplementary Information [file 41467_2018_5482_MOESM1_ESM.pdf]

Nucleoside-modified mRNA immunization elicits influenza virus hemagglutinin stalk-specific antibodies

Pardi et al.

Supplementary figures

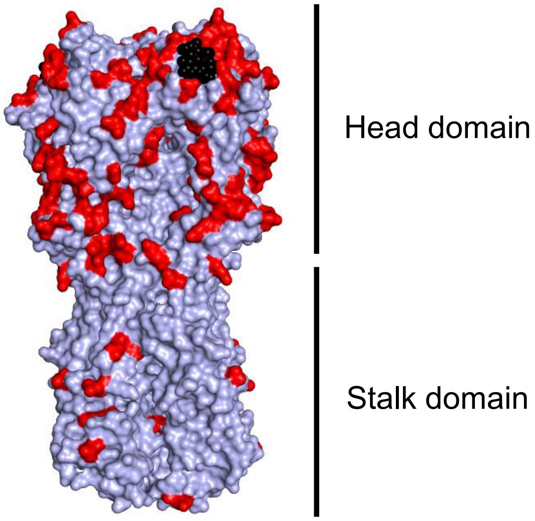

Supplementary Figure 1 Hemagglutinin structure and sequence conservation between the A/California/07/2009 and Puerto Rico/8/1934 influenza viruses. Shown is a structure of the hemagglutinin glycoprotein with the globular head and stalk domains denoted. Amino acid differences between A/California/07/2009 and Puerto Rico/8/1934 are highlighted in red on the A/California/07/2009 crystal structure (PDB ID 3UBN), while the cellular receptor sialic acid is shown in black.

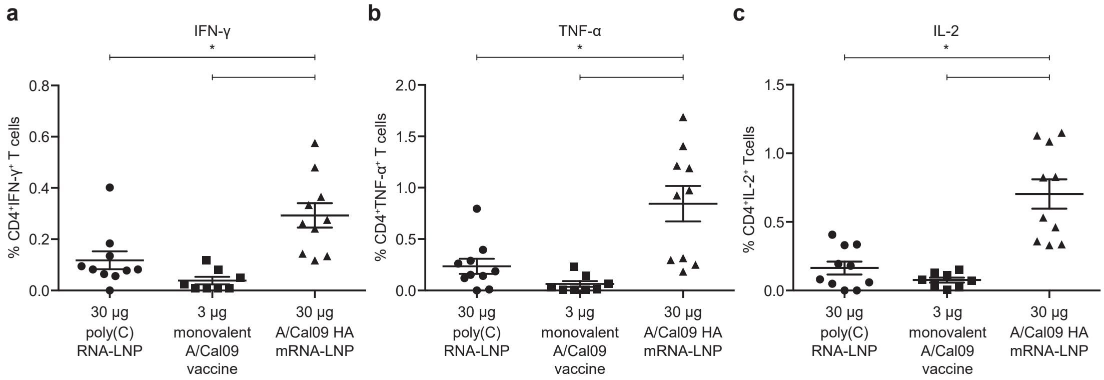

Supplementary Figure 2 Nucleoside-modified A/California/07/2009 HA mRNA-LNP immunization elicits antigen-specific $CD4^+$ T cell responses. Mice were immunized i.d. with a single dose of 30 µg of A/California/07/2009 HA mRNA-LNPs or 30 µg of poly(C) RNA-LNPs or i.m. with 3 µg of monovalent A/California/07/2009 virus vaccine. Splenocytes were stimulated with HA peptides 12 days after immunization and cytokine production by $CD4^+$ T cells was assessed by flow cytometry. Percentage of $CD4^+$ T cells producing (**a**) IFN-γ, (**b**) TNF-α and (**c**) IL-2 is shown. *n=8-10* mice and each symbol represents one animal. Experiments were repeated to achieve statistical significance. Horizontal lines show the mean. Error bars are SEM. Statistical analysis: one-way ANOVA with Bonferroni correction, *p<0.05

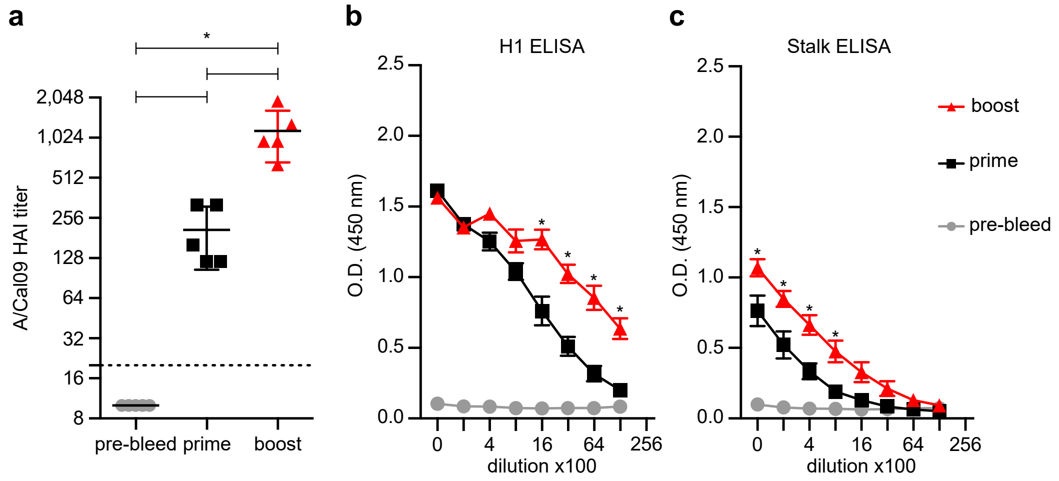

Supplementary Figure 3 Nucleoside-modified A/California/07/2009 HA mRNA-LNP immunization elicits HA stalk-reactive antibodies in rabbits. Animals were immunized i.d. two times with 50 µg of A/California/07/2009 HA mRNA-LNPs at week 0 and 6. Serum was collected at weeks 0 (pre-bleed), 6 and 18 and (**a**) HAI titers against the A/California/07/2009 virus, binding to full-length H1 HA (**b**) and cH6/1 HA (**c**) were determined. *n=5* rabbits and on (**a**) each symbol represents one animal. (**a**) Horizontal lines show the mean; dotted line indicates the limit of detection. (**a-c**) Error bars are SEM. Statistical analysis: (**a**) one-way ANOVA with Bonferroni correction, *p<0.05; (**b,c**) two-way ANOVA with Bonferroni correction, comparing post prime samples to post boost samples for each serum dilution *p<0.05.

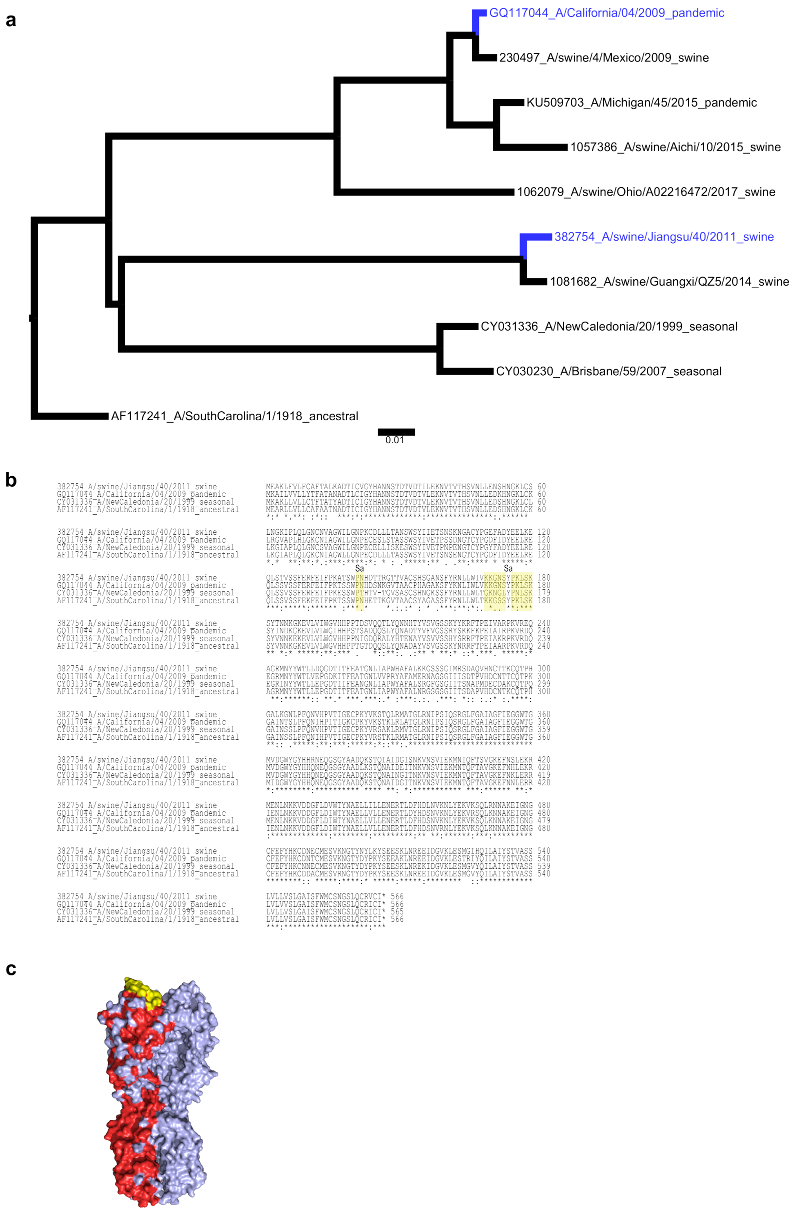

Supplementary Figure 4 Illustration of shared antigenic residues of pH1N1 and asH1N1. (**a**) Phylogenic tree based on the amino acid sequence similarity between the listed strains. A/California/04/2009 (pH1N1) and A/swine/Jiangsu/40/2011 (asH1N1) are highlighted in blue. The scale bar represents 1% divergence in amino acid sequence. (**b**) Multiple sequence alignment of the ancestral H1N1 strain (A/South Carolina/1/1918), pH1N1, and asH1N1. The Sa head antigenic site is highlighted in yellow to illustrate the 100% conservation between pH1N1 and asH1N1 at this site. (**c**) Three dimensional structure of the A/California/04/2009 (PDB ID 3LZG) HA protein with residues that are shared (100% conserved) between pH1N1 and asH1N1 shown on a single monomer in red. The Sa head antigenic site in yellow to show its position on the HA.

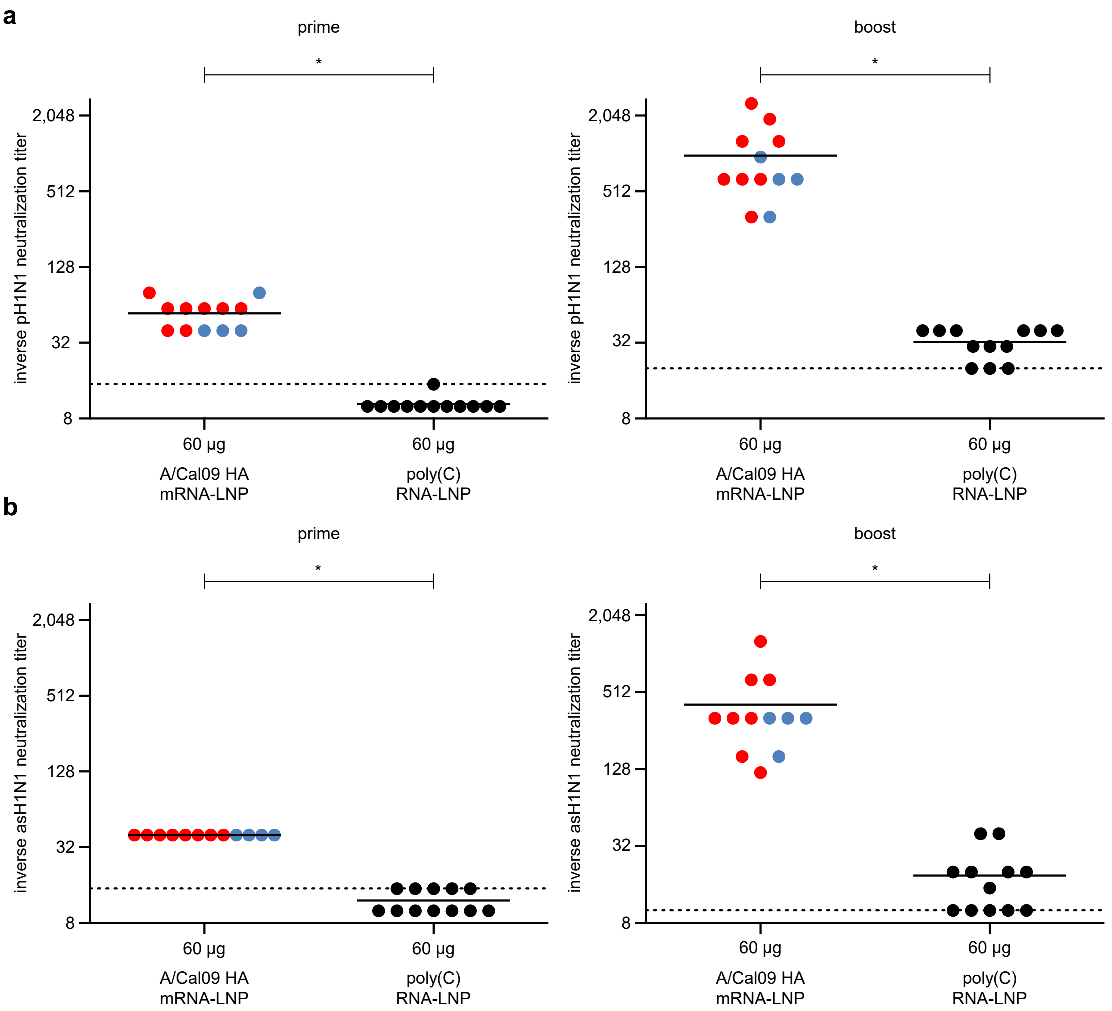

Supplementary Figure 5 Nucleoside-modified A/California/07/2009 HA mRNA-LNP immunization induces cross-reactive neutralizing antibodies in ferrets. Animals were immunized i.m. two times with 60 µg of A/California/07/2009 HA mRNA-LNPs or poly(C) RNA-LNPs at week 0 and 4. Serum was collected at weeks 0 (pre-vaccination), 4 and 13, and microneutralization titers against (**a**) pH1N1 and (**b**) asH1N1 influenza viruses were determined. Values are expressed as inverse microneutralization titers. Animals with no measurable A/California/07/2009 HAI activity after the first immunization are marked with blue dots. *n=12* ferrets and each symbol represents one animal. Horizontal lines show the mean; dotted line indicates the titer of the pre-vaccination serum pool. Statistical analysis: unpaired *t*-test comparing A/California/07/2009 HA mRNA-LNP-immunized animals to poly(C) RNA-LNP-immunized animals. *$p<0.05$
